# The Young Man's Opportunity

**Published:** 1920-01-17

**Authors:** 


### THE YOUNG MAN'S OPPORTUNITY.

THE last opening of a new medical session was most remarkable for the very great number of students then entering our hospitals, and since that time we have seen repeated reminders of coming congestion in the medical ranks. At the outset of this year we find, indeed, not only crowded schools, but waiting-lists of both male and female would-be doctors, and true it is that these circumstances should be given more than a passing thought before committing a member of the present student generation to a medical career. At all times medicine has been viewed generally as suitable only to those young people of reasonably acute scholastic powers, and if we admit that this requirement is now indeed intensified, we must also realise that he who joins our profession to-day begins under the ægis of a brand-new Government Department, designed—and likely to provide—both for the people an efficient and orderly medical service and for that service better means of discharging their important functions.

Though he may encounter greater competition, the young man will also undoubtedly meet with greater opportunities—an advance in both extent and variety on those of twenty years ago. And so to the large number of junior members of the profession who are either qualified and still at a loose end in the country, and the possibly greater number who are finding the greatest difficulty in their plan of action to follow approaching qualification, we would point out that this New Year is one in which, above all others, the young man's chance should occur.

The hardships experienced by many recently demobilised doctors are undoubted, but with these very sufferers lies the remedy. The helplessness of medicine as an organised calling demands united action. Some genuine form of union is urgently needed, and the right medical view and the reasonable public claim must be adjusted to the common satisfaction.

What are then the possibilities? At least a certain number of administrative posts will come into being, carrying regular salaries and graded to both time and responsibility. Panel practice itself will, in its imminent reorganised form, bring to the young man far greater remuneration than was ever offered to his father or grandfather if they followed the same paths in life. But such initial prosperity is far from the ideal. There is the great condemnation that while the start is so alluring the future holds but little greater promise. A panel income can increase only by increase of the size of the panel, and even though, for physical reasons, this may be an impossibility, for both public and scientific considerations it is certainly most undesirable. One thing at least is certain, and that is that general practice in Great Britain is, according to all old-time standards, largely in the melting-pot. This may not benefit all of us, but at least the change from our former routine will go hand-in-hand with added opportunity, not only in specialisation, but in every branch of our profession. At the moment it is a matter for regret that during such times there is no really satisfactory form of medical organisation or representation which can effectively help in guiding a representative medical policy. Nevertheless, though the younger man may even so have his opportunity in the end, he must remember that to view so pressing a matter with relative apathy, while those already established tend, some perhaps with their own axe to grind, to wrangle over unimportant side issues, is a course which will at least delay, if not altogether defeat, the efficiency of reform for which all are anxious. It is not alone the leaders of medical thought who must bestir themselves, but the profession as a whole, and particularly the rising generation. Then opportunity *must* come as their reward.

